# Transcriptome analysis of ovarian cancer uncovers association between tumor-related inflammation/immunity and patient outcome

**DOI:** 10.3389/fphar.2025.1500251

**Published:** 2025-02-06

**Authors:** Jingfang Wang, Wenrui Zhu, Xia Li, Yuanyuan Wu, Wenhui Ma, Yangzhou Wang, Weihong Zhao, Fang Wei, Wenhao Wang

**Affiliations:** ^1^ Department of Obstetrics and Gynecology, Second Hospital of Shanxi Medical University, Taiyuan, Shanxi, China; ^2^ Department of Stomatology, Changzhi Medical College, Changzhi, Shanxi, China

**Keywords:** epithelial ovarian cancer, tumor immunotherapy, tumor immune microenvironment, cancer prognosis model, tumor-associated macrophages (TAM)

## Abstract

**Background:**

Epithelial ovarian cancer (EOC) is a cancer that affects the female reproductive system and is highly lethal. It poses significant challenges in terms of treatment and often has a poor prognosis. In recent years, with the advent of PARPi, the treatment of ovarian cancer has entered a new stage of full-process management. Although more and more drugs have been approved, the therapeutic effect of PARPi is still very limited. With the rapid development of PD-1/PD-L1, CTLA-4, oncolytic viruses, cancer vaccines, adoptive cell therapy, etc., tumor immunotherapy has provided new opportunities for the treatment of ovarian cancer.

**Methods:**

This study used comprehensive transcriptome analysis across multiple databases to gather gene transcripts and clinical features of normal ovarian samples and tissue samples from ovarian cancer. The aim was to explore the mechanisms underlying tumor immunotherapy resistance and to reveal the relationship between ovarian cancer’s immune microenvironment and genes linked to inflammation. Various R packages were used for differential gene analysis, enrichment analysis, co-expression network construction, and prognostic model building.

**Results:**

It has been found that the prognosis of ovarian cancer patients is closely associated with sets of genes involved in inflammation. The immune infiltration microenvironment, clinicopathological features, and survival rates differed significantly between two inflammatory gene expression patterns identified using cluster and immune microenvironment analyses. Further analysis revealed that the high-risk group had a higher abundance of M2-type macrophage infiltration, more active anti-tumor immune response, higher tumor stemness score, potentially worse prognosis, and lower response rates to multiple chemotherapy drugs and immune checkpoint inhibitors.

**Conclusion:**

These findings provide new perspectives and potential targets for immunotherapy and prognostic evaluation of ovarian cancer and offer new strategies and directions for clinical treatment and patient management. This study provides crucial information to further our comprehension of drug response mechanisms and tumor immunotherapy. It offers new strategies and methods for the treatment and prognostic improvement of ovarian cancer.

## 1 Introduction

In the female reproductive system, the deadliest cancerous growth is called epithelial ovarian cancer (EOC). Ovarian cancer ranks seventh among malignant tumors in women globally, accounting for over 310,000 new cases annually, according to the 2020 Global Cancer Statistics (Lee et al., 2022; Konstantinopoulos and Matulonis, 2023). Every year, ovarian cancer claims the lives of about 210,000 people. In 2020, ovarian cancer was diagnosed in 60,000 new cases and killed 40,000 people in China (Zhao et al., 2023). Patients with advanced stage ovarian cancer have an approximately 30% 5-year survival rate. With multiple recurrences, the interval between treatments and recurrences becomes shorter, leading to decreased sensitivity to platinum-based drugs and eventually developing into platinum resistance. The treatment is highly challenging, and the prognosis is often poor (Marchetti et al., 2021; Porter and Matulonis, 2023). Overcoming chemotherapy resistance in ovarian cancer is an urgent and important clinical issue.

Inflammation reactions are mainly divided into acute and chronic types. Acute inflammation occurs mainly in physical, chemical, or acute infection conditions as the body’s early defense mechanism, and it usually resolves quickly on its own (Yang et al., 2023). Chronic inflammation, on the other hand, occurs in chronic infections or autoimmune diseases, where the body’s normal feedback regulation cannot stop the inflammation, leading to chronic inflammation (Liu et al., 2022). Statistics show that chronic inflammation contributes to about 20% of malignant tumors worldwide (Kennel et al., 2023; Venakteshaiah and Kumar, 2021; Haas et al., 2021). Non-steroidal anti-inflammatory drugs clinically reduce the incidence and metastasis of various solid tumors and decrease tumor-induced mortality. Chronic inflammation is thought to significantly influence the initiation, growth, and progression of cancers.

The mechanisms through which chronic inflammation initiates tumor occurrence, and development are diverse but often involve the microenvironment provided by inflammation for tumors. As a crucial part of the cancer stroma, cancer-associated fibroblasts (CAFs) are intimately associated with inflammation and the tumor immune microenvironment (TME) (Chen et al., 2021). CAFs interact with various signaling pathways such as NF-κB, PI3K-Akt, IL6-JAK-STAT3, and TGF-β to help form and maintain the TME, influencing ECM structure and generating immune therapy resistance (Mao et al., 2021; Wu F. et al., 2021). Additionally, activated CAFs promote monocyte adhesion and drive macrophages toward M2 polarization, further inhibiting immune responses in the TME (Lavie et al., 2022; Galbo et al., 2021). Therefore, analyzing the relationship between genes linked to inflammation and the tumor immune milieu can aid in comprehending reasons for EOC immunotherapy resistance and contribute to developing innovative immunotherapy strategies.

## 2 Methods and materials

### 2.1 Data acquisition

The TCGA database (https://portal.gdc.cancer.gov/) included the gene transcripts and clinical details of 429 ovarian cancer tissue samples from patients with the disease. The patient’s clinical features encompassed survival status, time, tumor grade, age, etc. In the meantime, the GTEx database (https://www.gtexportal.org/home/) was accessed to download 88 normal ovarian samples. For validation, the gene expression profiling microarray datasets for ovarian cancer tissues were acquired from the GEO database (https://www.ncbi.nlm.nih.gov/geo). These datasets, GSE26712 (Zheng et al., 2019) and GSE102073 (Ye et al., 2021), each contained 153 and 84 ovarian cancer tissues, respectively. Additionally, ovarian cancer single-cell datasets EMTAB8107 (Ding et al., 2024), GSE118828 (Yu et al., 2022), GSE130000, and GSE154600 (Jiang et al., 2023) were downloaded to explore gene expression at the single-cell level.

### 2.2 Acquisition of inflammation-related gene sets

Inflammation-related gene sets were obtained from the Molecular Signatures Database (MSigDB) (Castanza et al., 2023) (https://www.gsea-msigdb.org/gsea/msigdb/), including BIOCARTA_INFLAM_PATHWAY (v2023.2.), GOBP_CHRONIC_INFLAMMATORY_RESPONS (v2023.2), HALLMARK_INFLAMMATORY_RESPONSE (v2023.2.), and REACTOME_INFLAMMASOMES (v2023.2.).

### 2.3 Scoring of inflammation-related gene sets and prognostic evaluation

With the help of the GSVA (Hänzelmann et al., 2013) R package (v2.0.4), we evaluated the gathered sets of gene sets associated with inflammation using single-sample gene set enrichment analysis (ssGSEA). ssGSEA is an extension of the GSEA method, primarily designed for individual samples where GSEA is not applicable. The algorithm uses the empirical cumulative distribution function to calculate enrichment scores (ES) and rank normalized gene expression values for a given sample. The prognostic correlation between gene sets associated with inflammation and patients with ovarian cancer was assessed simultaneously using the Cox proportional hazards model.

### 2.4 Consensus clustering based on inflammation-related gene set scores

Consensus clustering was often used in cancer subtype classification studies using the Consensus ClusterPlus R package (v4.12.6) (Wilkerson and Hayes, 2010). This study’s ovarian cancer subtype classification was conducted based on the aforementioned inflammation-related gene set scores. The optimal clustering effect was determined by combining the consensus cumulative distribution function (CDF) with the Proportion of Ambiguous Clustering (PAC) score. In the CDF plot, the consensus matrix’s cumulative distribution function was displayed for different values of k (represented by colors), aiding in identifying the approximate maximum CDF value, where consensus and cluster confidence are maximized, resulting in the most reliable clustering analysis. In PAC analysis, lower PAC values indicate more ideal clustering effects.

### 2.5 Kaplan-Meier (KM) survival analysis

Currently, the most widely used method for survival analysis is the Kaplan-Meier approach. The KM approach, as it is commonly called, was proposed by Kaplan and Meier. The Kaplan-Meier survival analysis compares the survival circumstances of two patient groups using a univariate analysis that integrates patients’ survival times and terminal states. The Kaplan-Meier survival curve, a commonly encountered representation, visually reflects survival differences under various conditions.

### 2.6 Immune cell infiltration analysis

Based on the IOBR (Zeng et al., 2021) R package (v2.0), we employed built-in algorithms such as TIMER, CIBERSORT, MCPcounter, EPIC, and quanTIseq (Newman et al., 2015; Becht et al., 2016; Finotello et al., 2019; Racle et al., 2017; Li et al., 2016) to assess the abundance of immune cell infiltration in the tumor immune microenvironment of each ovarian cancer tissue.

### 2.7 Drug sensitivity analysis

The OncoPredict (Maeser et al., 2021) R package (v1.2) was created by Maeser et al. and was used to predict medication reactions in cancer patients. OncoPredict adapts tissue gene expression patterns to the semi-maximal inhibitory concentration (IC50) of drugs taken from cancer cell lines in the Genomics of Drug Sensitivity in Cancer (GDSC) database and the Cancer Cell Line Encyclopedia (CCLE) maintained by the Broad Institute. An unpaired *t*-test was used to assess the sensitivity of 198 drugs (between high-risk and low-risk groups). Set at p < 0.05 was the significance level.

### 2.8 Prediction of immunotherapy sensitivity

The Cancer Immunome Atlas (TCIA) (Charoentong et al., 2017) database (https://tcia.at/) was used to download the Immunophenotype Scores (IPS) for CC. Subsequently, IPS were compared across different tumor groups to predict sensitivity to immunotherapy.

### 2.9 Differential gene identification

Using the limma (Ritchie et al., 2015) R package (3.60.4), differential gene expression analysis was performed on the TCGA data. This involved data preprocessing, normalization, and identifying significant differences in gene expression levels through linear modeling. Statistical thresholds were set (adj. P. Val. < 0.01 and |log2(FC)| > 1) to screen for significantly differentially expressed genes. Finally, to decipher the biological significance of these differential genes, enrichment analysis and functional annotation were performed.

### 2.10 Enrichment analysis

The clusterProfiler (Wu T. et al., 2021) R package (v4.12.6) was used to perform enrichment analysis, which consisted of two steps: (1) Over-Representation Analysis (ORA) to investigate the functional enrichment of gene sets through Genomes (KEGG) analyses, Kyoto Encyclopedia of Genes, Gene Ontology (GO), and (2) Gene Set Enrichment Analysis (GSEA) to examine the enrichment of validated gene sets in KEGG pathways. These enrichment results revealed the gene sets’ biological functions and pathway associations.

### 2.11 Weighted gene Co-Expression network construction

In systems biology, co-expression gene modules and their correlation to phenotypes are identified using Weighted Gene Co-Expression Network Analysis (WGCNA) (Langfelder and Horvath, 2008). Gene co-expression networks are constructed by calculating gene-gene correlations and then converting the correlation matrix into a weighted matrix. Gene modules are then built based on the weighted matrix, and the eigengene for each module is computed. Subsequently, the correlation between module eigengenes and phenotypic data determines the module-phenotype relationship. Ultimately, gene modules associated with phenotypes of interest are identified, revealing underlying biological pathways and mechanisms. The WGCNA method aids in uncovering key modules and gene correlations within gene regulatory networks.

### 2.12 Prognostic model construction and validation

The TCGA dataset was initially used to construct a survival prognostic model using the multiCox and Least Absolute Shrinkage and Selection Operator (LASSO) techniques. Gene features were selected using LASSO, and multiCox was employed for multivariable Cox regression to establish the prognostic model. The model was then used for independent external validation datasets, and its ability to predict survival was evaluated using Kaplan-Meier survival analysis and time-dependent ROC curve analysis. These validation analyses verified the prognostic predictive efficacy of the model across different datasets, ensuring its reliability and generalizability.

### 2.13 mRNA Stemness Index (mRNAsi)

Derived from the PCBC database’s mean-centered RNA-Seq data of PSCs (syn2701943) (Gul et al., 2023). A stem cell feature signature was found using the One-Class Logistic Regression (OCLR) machine learning approach, and it was confirmed using leave-one-out cross-validation. A Spearman correlation analysis was then used to compare the stem cell features and the normalized expression matrix of tumor samples. Finally, by scaling the Spearman correlation coefficient between 0 and 1, the mRNA Stemness Index (mRNAsi) was determined. A higher mRNAsi indicates a higher degree of tumor dedifferentiation and stronger stemness.

### 2.14 Single-cell analysis

The following methods were used to process ovarian cancer single-cell sequencing data: We first converted the scRNA-seq data into a Seurat (Hao et al., 2024) object using the Seurat R package. We performed quality control (QC) by determining the percentage of ribosomal or mitochondrial genes and eliminating low-quality cells. FindVariableFeatures was used to determine the top 2000 genes exhibiting high variability. Furthermore, dimensionality reduction techniques were used to group approximately 2000 genes using Principal Component Analysis (PCA) and Uniform Manifold Approximation and Projection (UMAP). We could identify marker genes for each cluster using the FindAllMarkers tool with |Log2FC| and min. 0.3 and 0.25 are the respective pct cutoff values. Different cell types were annotated using the SingleR (Aran et al., 2019) R package. Finally, we used the AddModuleScore function to compute the expression levels of prognostic model genes at the single-cell level.

### 2.15 Cell lines

Fenghuishengwu in China is where human ovarian surface epithelial cells (HOSE) are sourced (HOSE, CL0154). From the American Type Culture Collection, the SKOV3 cell line was acquired. *Mycoplasma* is routinely tested for in all cell lines. A complete medium, consisting of 1% double antibiotics and 10% fetal bovine serum (FBS), is used to cultivate both HOSE and SKOV3. When the cell confluence reaches 80%–90%, they are passaged.

### 2.16 Cell transfection

Similarly, 5 × 10^5^ cells were cultured in each well of a 6-well plate. For transfection, HOSE and SKOV3 cells were treated with 15 nM of siRNA IL-6, siRNA TGF-β1 and siRNA NC (P4157, GenePharma, Shanghai, China), respectively, using Lipofectamine 3000 (L3000150, Thermo, New York, Waltham, MA, United States).

### 2.17 qPCR

HOSE and SKOV3 cells were lysed to obtain total RNA using a TRIzol reagent (15,596,026, Invitrogen, New York, NY, United States). The qRT-PCR analysis was performed using the HiScript II One Step qRT-PCR SYBR Green kit (P131, Vazyme, Nanjing, China) and a Bio-Rad CFX96 PCR system (Bio-Rad, Hercules, CA, United States). RuiBiotech (Beijing, China) created and manufactured the primers used in this investigation. Glyceraldehyde-3-phosphate dehydrogenase (GAPDH) was used as the internal reference for assessing the target gene expression using the 2^−ΔΔCT^ method.

### 2.18 Western blot

The total protein was extracted using a cell lysate solution, and the proteins were separated using 10% sodium dodecyl sulfate-polyacrylamide gel electrophoresis (SDS-PAGE). PVDF membranes were then used to hold the separated proteins (03010040001, Millipore, Billerica, MA, United States). After a 30-min blocking, the membranes were subjected to adding primary antibodies and incubated at 4°C. The primary antibodies were obtained from Abcam (Abcam, United States). Secondary antibodies (BA1054, 1:5000, Boster, Wuhan, China) were added to the membranes and incubated for 2 h at room temperature after washing. The membranes were then visualized using an ECL development kit (A38554, Invitrogen, New York, NY, United States) and photographed with a GE Las-4000 (GE Healthcare, Piscataway, NJ, United States). After conducting the experiment thrice, the gray values were obtained using Media Cybernetics’ ImageJ 1.8.0 program (Silver Spring, MD, United States). An internal reference was beta-actin.

### 2.19 Statistical analysis

The analysis was conducted using SPSS 26.0 and the R programming language. The measurement data was expressed using the standard deviation (x ± s). A one-way ANOVA was employed to compare the groups. Dunnett’s multiple comparisons were performed to determine whether the variance was uneven. Measurement data with a normal distribution were displayed as mean ± standard deviation, and t-tests were used to compare groups. The Mann-Whitney *U* test was performed using measurement data that was not normally distributed and displayed as the median and interquartile range to compare groups. Count data were expressed as rates, and group comparisons were conducted using the χ2 test.

## 3 Results

### 3.1 Identification of inflammatory molecular subtypes in ovarian cancer based on consensus clustering

**FIGURE 1:**
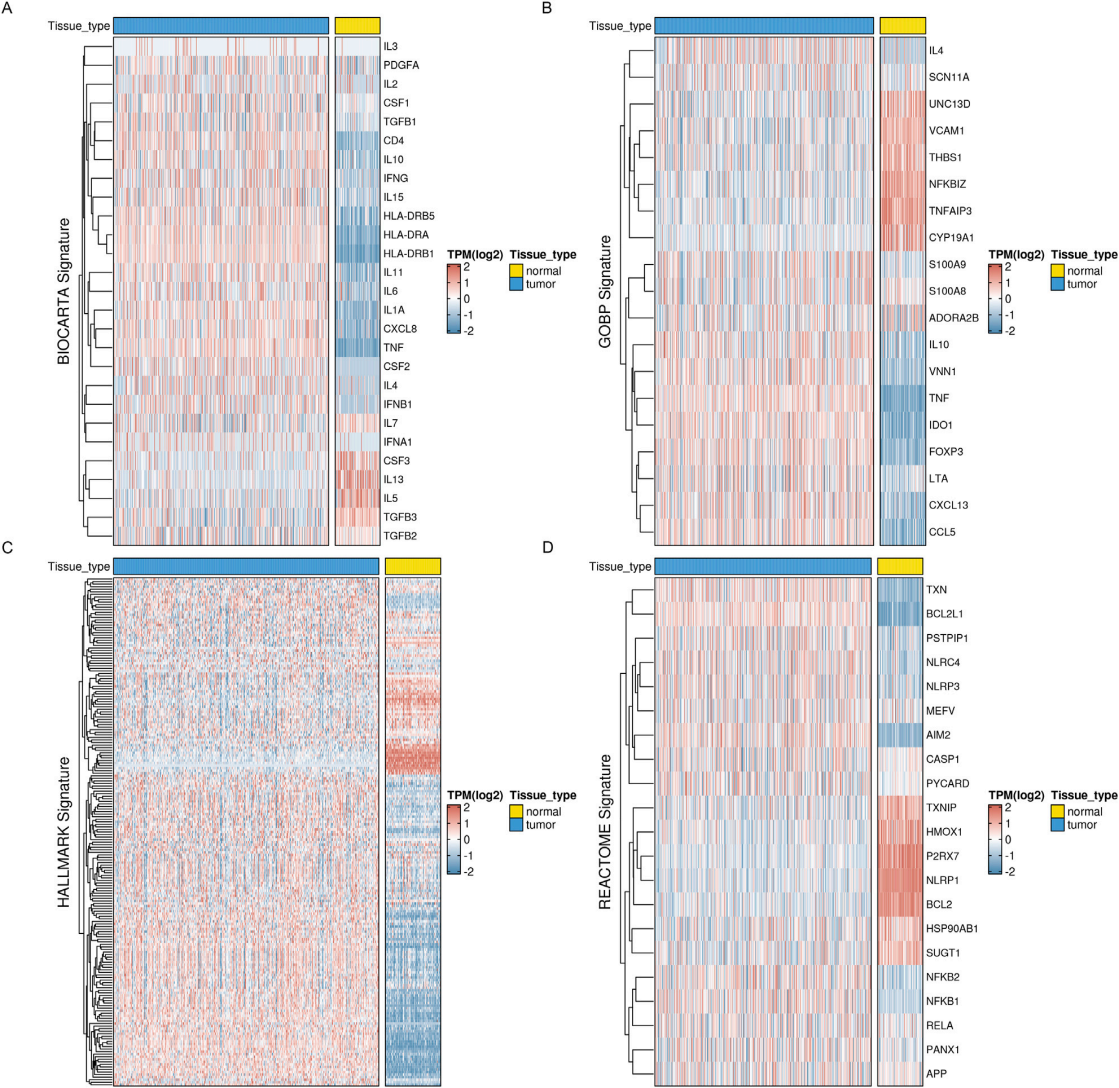
Expression profiles of inflammation-related markers in OV reveal distinct patterns. Four key inflammation-related signatures, such as the BIOCARTA_INFLAM_PATHWAY, have been examined for their expression levels **(A)**, GOBP_CHRONIC_INFLAMMATORY_RESPONS **(B)**, HALLMARK_INFLAMMATORY_RESPONSE **(C)**, and REACTOME_INFLAMMASOMES **(D)**.

First, we obtained four inflammatory-related gene sets from the MSigDB database, including BIOCARTA_INFLAM_PATHWAY, GOBP_CHRONIC_INFLAMMATORY_RESPONS, HALLMARK_INFLAMMATORY_RESPONSE, and REACTOME_INFLAMMASOMES. Most inflammatory-related genes had significantly different expression profiles in tumor and normal tissues, with most genes significantly elevated in tumor tissues, according to our analysis of the TCGA ovarian cancer dataset and the corresponding normal tissues from the GTEx database (Figure 1). This suggests a correlation between inflammatory phenotypes and tumor development.

**FIGURE 2:**
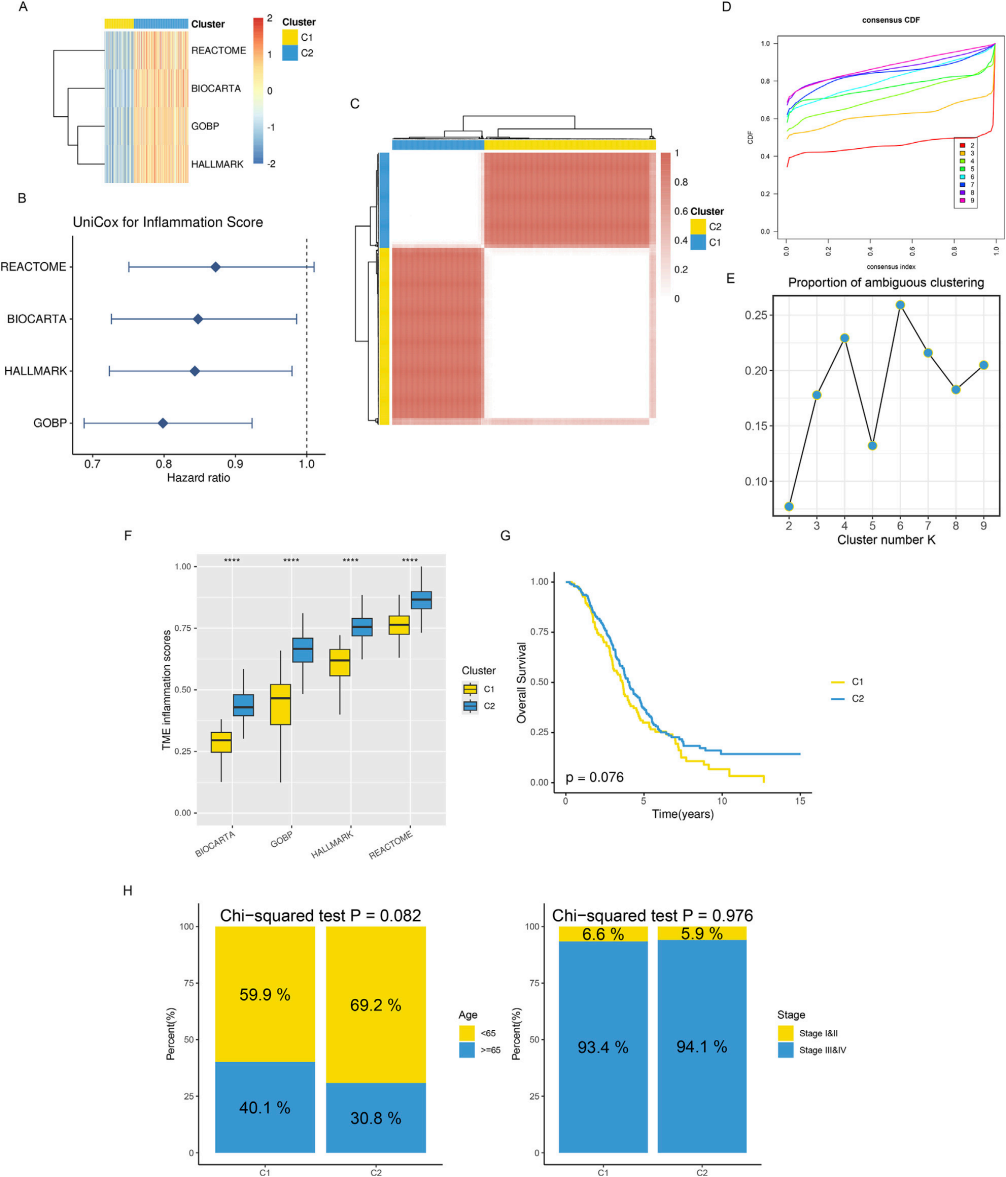
Distinct TME landscapes in OV. **(A)** The GSVA score of each signature is associated with inflammation between two subclusters. **(B)** A forest plot displaying the hazard ratio for each signature associated with inflammation was found using univariate Cox regression analysis. **(C)** The TCGA-OV consensus score matrix for the glioma sample, with k = 2 clustering number. The consensus score indicates the degree of interaction between two samples. **(D, E)** The consensus matrix’s PAC scores **(E)** and CDF curves **(D)** for each **(K) (F)** Boxplots that display the distribution of GSVA scores for every inflammatory signature between two subclusters; **(G)** Kaplan-Meier curves that analyze survival differences between two subclusters using the log-rank test. **(H)** Stacked bar graphs showing the distributions between two subclusters for age populations (left panel) and stages (right panel). Using Chi-squared testing, P values were calculated.

Meanwhile, based on ssGSEA, we performed enrichment scoring of inflammatory-related gene sets for each ovarian cancer tissue (Figure 2A). Univariate COX regression analysis identified three inflammation-related gene sets as significantly and positively associated with better prognosis (HR < 1) in ovarian cancer patients. These gene sets are thought to be protective prognostic factors (Figure 2B). This suggests these inflammatory-related gene sets’ potential research value and clinical significance. We, therefore, performed consensus clustering analysis using the Consensus ClusterPlus R package based on the enrichment scores of these four inflammatory-related gene sets. The maximum number of clusters was set to 10, with 100 subsamples drawn, a sample proportion of 0.8, K-means as the clustering algorithm, and Euclidean distance as the metric. Ultimately, we performed nine clusters with k values ranging from 2 to 10. Through comprehensive evaluation using CDF curves and PAC analysis, we selected the ideal number of clusters as 2 (Figures 2C–E). At the same time, we found significant differences in inflammatory enrichment scores (Figure 2F) and overall survival rates (Figure 2G, log-rank p = 0.076) among patients with different inflammatory gene expression patterns. Chi-square tests for clinicopathological features revealed differences in the age distribution (with 65 years as the cutoff) and clinicopathological grading among patients in different groups (Figure 2H).

### 3.2 Distinct immune infiltration microenvironments, responsiveness to drug treatment, and deregulated signaling pathways among subtypes

**FIGURE 3:**
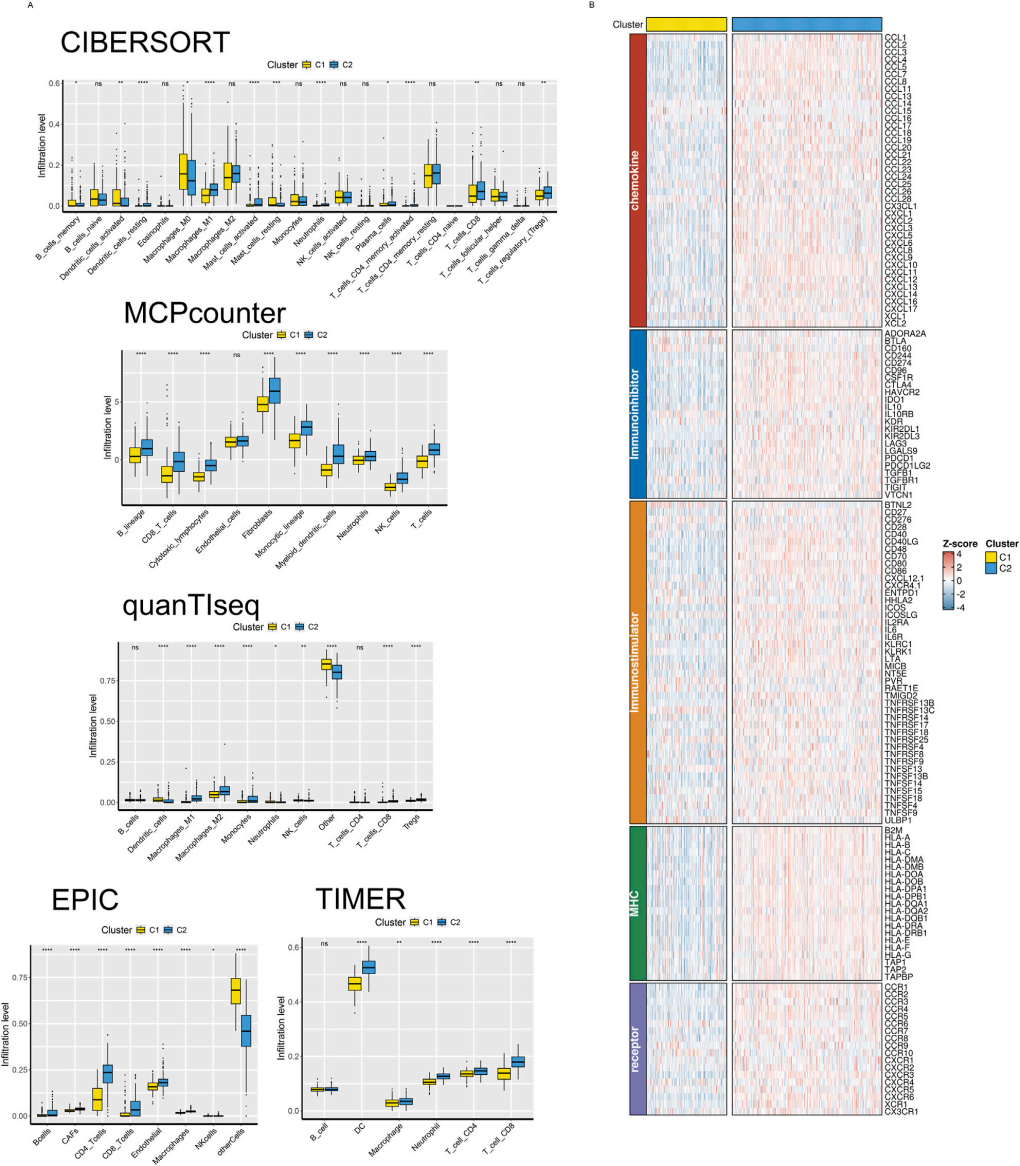
A hot-TME is shaped by the C2 subcluster in OV. **(A)** The immune cell subset infiltration abundances for two TME subclusters were measured using CIBERSORT, MCP-counter, quanTIseq, EPIC, and TIMER. **(B)** The patterns of immunoregulator expression for each of the two TME subclusters.

Previous studies have reported that different tissue types often have distinct immune infiltration microenvironments. Thus, we used five immune microenvironment analysis methods in this study—CIBERSORT, MCPcounter, quanTIseq, EPIC, and TIMER—for integrated evaluation and analysis of immune cell infiltration profiles to thoroughly examine the immune profiles among various subtypes. We found that the C2 subtype had much more infiltrating NK cells, B cells, macrophages, CD8^+^ T cells, and CD4^+^ T cells than the C1 subtype, with consistent results across various analysis methodologies. This suggests that the C2 subtype exhibits the biological characteristics of a so-called “hot” tumor immune microenvironment (Figure 3A). In the meanwhile, we found that the C2 subtype had far higher amounts of immunomodulators and cytokines expressed than the C1 subtype, based on a list of genes encoding immunomodulators and chemokines that we downloaded from the TISIDB database (Figure 3B).

**FIGURE 4:**
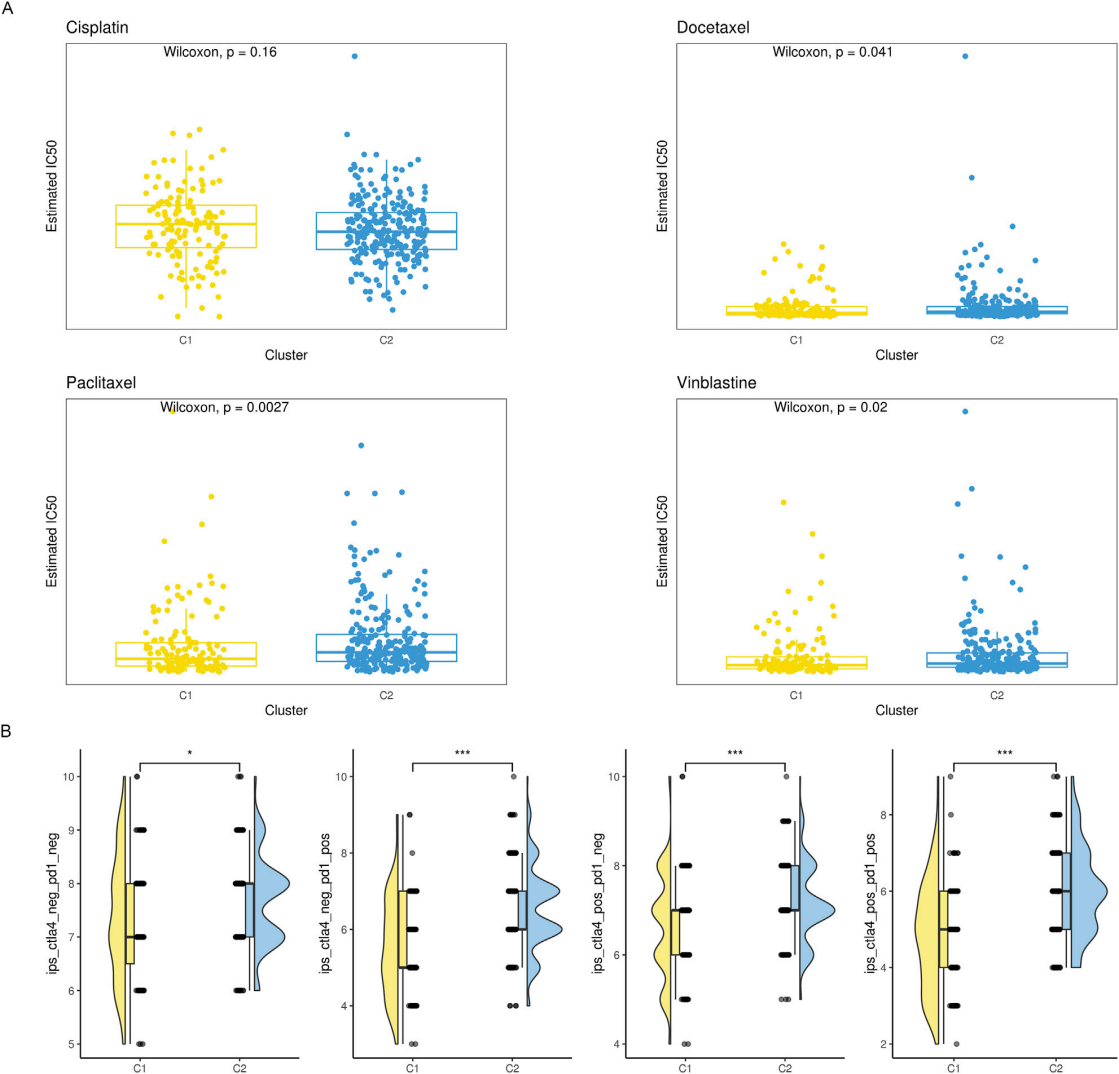
The drug sensitivity of the two subgroups. **(A)** Violin plot showing the estimated half-life (IC50) of chemotherapy drugs between two subgroups. **(B)** A raincloud plot that shows the difference in IPS scores between two subclusters.

With the deepening of research on the immune tumor microenvironment, substantial evidence suggests that tumors with different levels of immune cell infiltration have distinct response rates to chemotherapy and immunotherapy. Therefore, we examined the IC50 values of vinblastine, paclitaxel, docetaxel, and cisplatin. We found that, except cisplatin, the C2 subtype’s IC50 values were considerably higher than the C1 subtype’s, indicating a noticeably lower response rate of the C2 subtype to these three drugs (Figure 4A). Simultaneously, we utilized IPS, IPS-PD1/PD-L1/PD-L2, IPS-CTLA4, and IPS-PD1/PD-L1/PD-L2 + CTLA4 to assess differences in the response rates to immune checkpoint inhibitor therapy among different subtypes. We found that IPS, IPS-PD1/PD-L1/PD-L2 and IPS-CTLA4 were significantly higher in the C2 subtype compared to the C1 subtype (Figure 4B).

**FIGURE 5:**
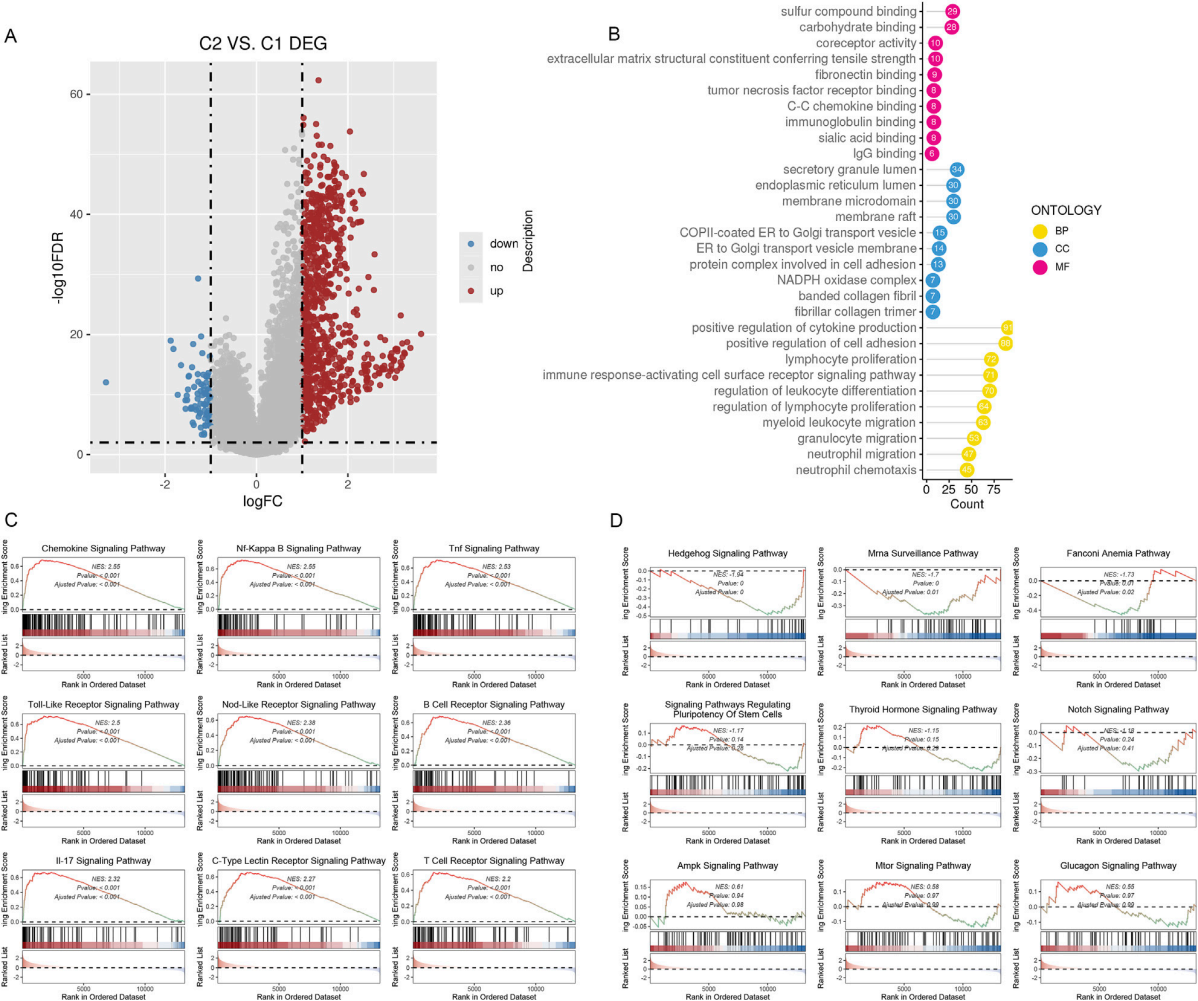
DEGs between the two subclusters. **(A)**The volcano map shows the genes classified as downregulated (blue) and upregulated (red) inside the two subclusters. **(B)** The 10 most gene-enriched GO terms in hubs. **(C, D)**: GSEA of the dysregulated pathways in the C2 subclusters.

We hypothesize that the distinct immune infiltration microenvironments and responses to drug treatment among different subtypes are based on significantly different signaling pathways and biological differences. Therefore, we identified genes that were differently expressed among various subtypes using the limma R package (Figure 5A). We carried out an over-representation analysis (ORA), which included GO_BP/CC/MF (Figure 5B), and last, we performed a GSEA analysis. The results suggested multiple signaling pathways were significantly deregulated in C2 (Figures 5C, D). In summary, subtypes based on inflammatory gene enrichment scores exhibit distinct immune infiltration microenvironments, responses to drug treatment, and deregulated signaling pathways, warranting further investigation.

### 3.3 Identification of biological features of different inflammatory subtypes using WGCNA gene co-expression network analysis

**FIGURE 6:**
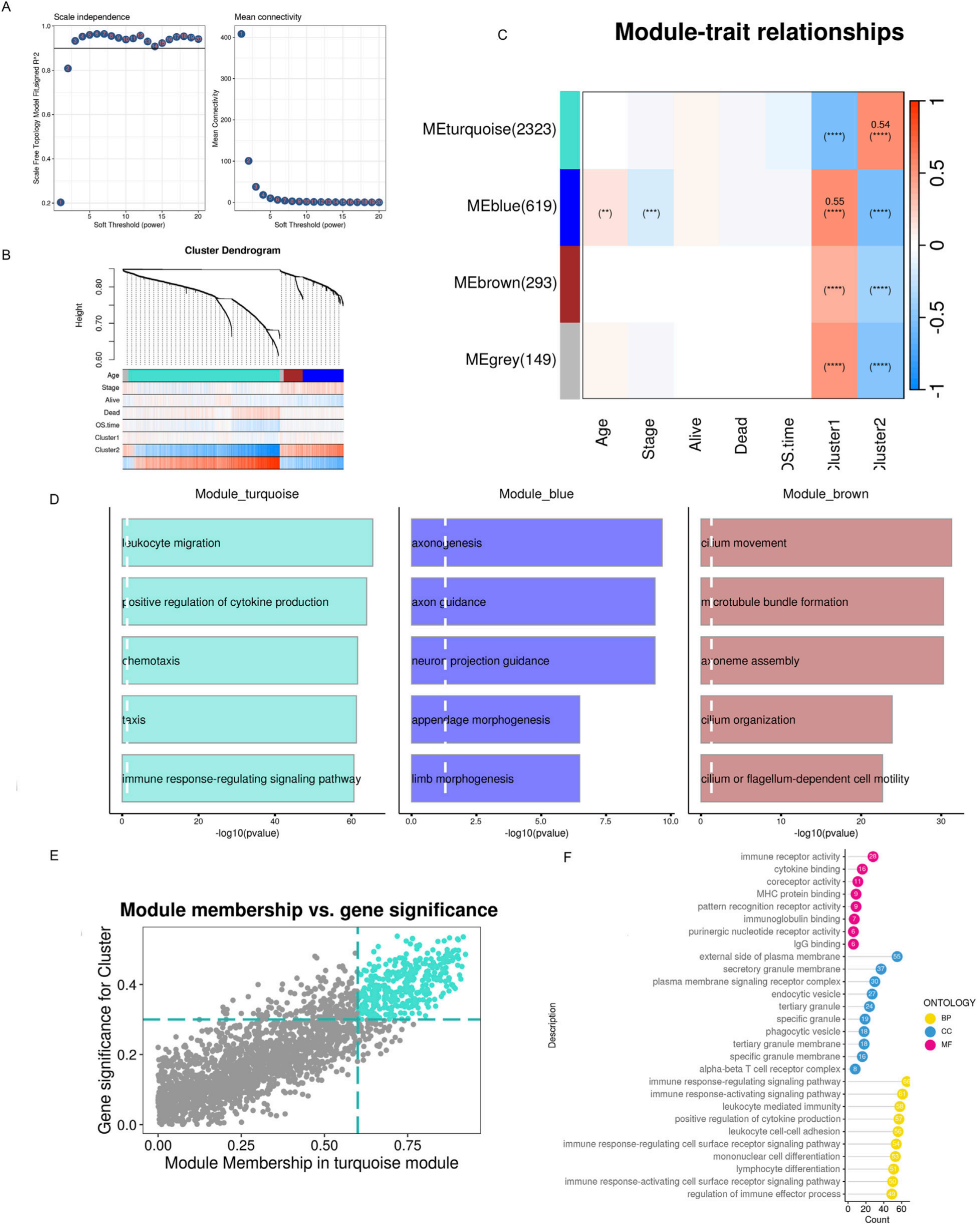
WGCNA detects modules associated with subclusters and hub genes embedded within them. **(A)** Examination of network configuration for various soft-threshold power levels. On the scale-free topology fit index, the left panel illustrates the effect of a soft-threshold power of 3. The effect of the same criterion on the average connectivity is shown in the right panel **(B)** Cluster dendrogram of the modules exhibiting coexpression. Each color corresponds to a co-expression module. **(C)** A module-trait heatmap shows how clinical traits and module eigengenes relate. **(D)** Bar charts showing the top five enriched phrases for every module gene. The connection between gene significance and module membership in the brown modules. **(E)** Hub genes of the appropriate module were identified as dots in color with MM > 0.6 and GS > 0.3. **(F)** Top 10 enriched GO terms of hub genes.

To conduct the WGCNA analysis, we first added the differentially expressed genes identified in the previous step. Four co-expression modules were obtained after setting the soft threshold β to three and the minimum number of genes in a module to 30 (Figures 6A, B). We utilized GO enrichment analysis and found that modules other than the gray module possessed distinct biological characteristics. Since previous analyses suggested that patients in the C2 subtype had a better prognosis, responsiveness to drug treatment, and deregulated signaling pathways, we hypothesized that genes significantly associated with C2 might be involved in tumor development, invasion, and resistance to drug treatment. Based on WGCNA co-expression network analysis, we found that the C2 subtype had the strongest positive correlation with the turquoise module (Figure 6C, Cor = 0.54), which contained 2323 genes. We could run functional enrichment analyses on these genes using thresholds (MM > 0.6 and GS > 0.3) to identify key genes inside the module (Figures 6D, E). The results showed that immune receptor activity and other biological processes were the primary roles of the module’s key genes (Figure 6F).

### 3.4 Construction of an ovarian cancer prognosis model based on inflammatory-related prognostic genes

The key genes found in the previous step served as the prognosis model’s input genes. In this study, we used the TCGA dataset to train the model. Then, we assessed the model’s prognostic efficacy using independent external datasets based on time-dependent ROC curves and Kaplan-Meier survival analysis.

**FIGURE 7:**
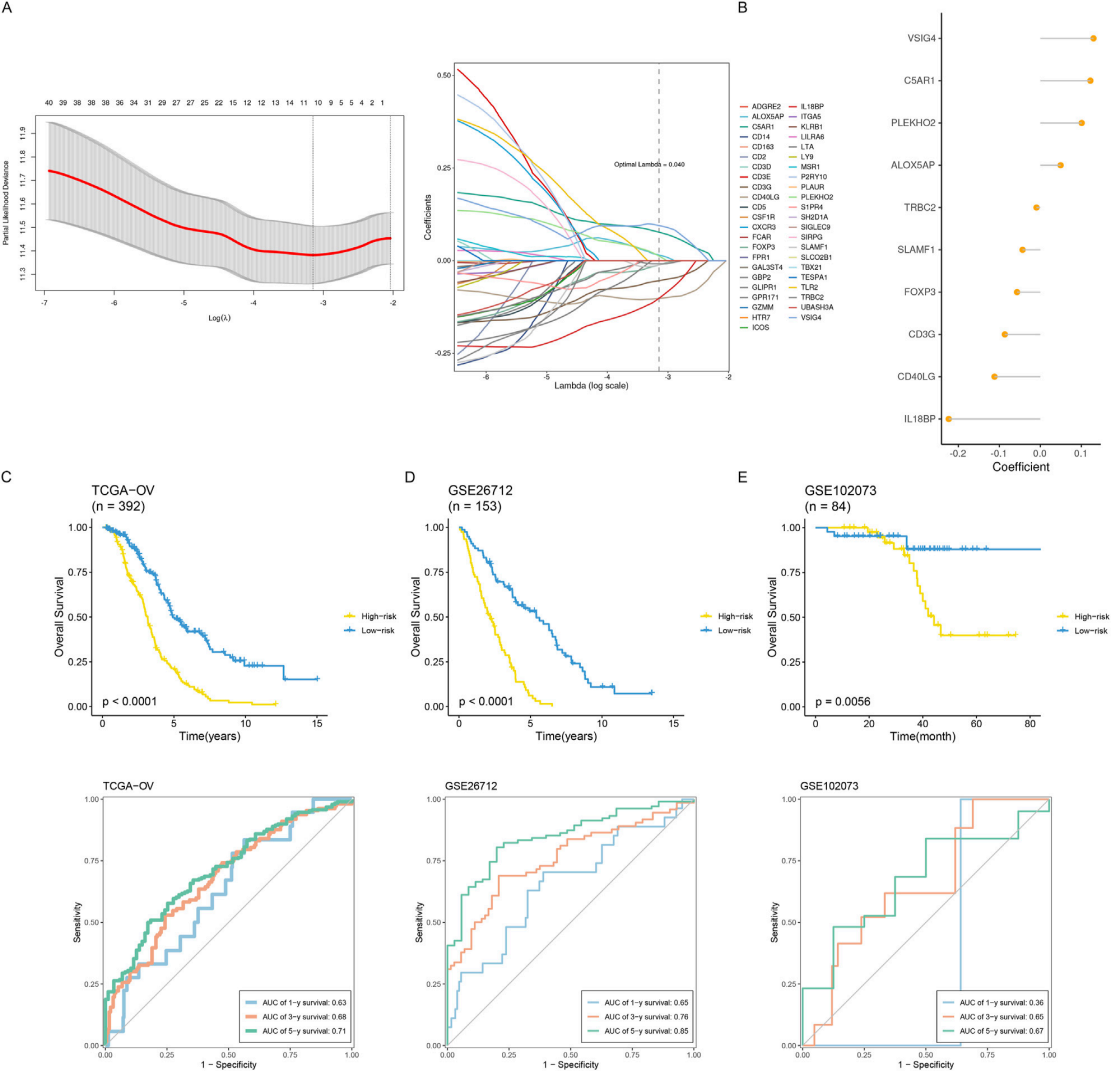
Construction and validation of an inflammatory prognostic signature. **(A)** Identification of prognostic hub genes using the optimal parameter (λ) obtained from the LASSO regression analysis. **(B)** The coefficients of signature genes are shown in a multiCox regression analysis-calculated lollipop chart. **(C–E)** Comparing how two groups’ survival rates differed throughout the three datasets. Time-dependent ROC examination of the three datasets’ model.

With the help of multivariate COX regression analysis, Least Absolute Shrinkage and Selector Operation (LASSO) (Figure 7A), and module key genes, we constructed a prognosis model in this study. The genes included in the model and their corresponding regression coefficients are shown (Figure 7B). Risk scores based on the prognostic model were simultaneously computed for all ovarian cancer samples in the training and validation sets. The samples were divided into low-risk and high-risk groups based on the median risk score. Our result shows the plotted Kaplan-Meier curves, which indicate substantial differences between the two groups (Figures 7C–E). Using a time-dependent ROC curve analysis, the prediction efficiency at 1, 3, and 5 years was also evaluated, and the results indicated that the prognostic model had good prediction performance.

### 3.5 Relationship between risk score and tumor immune microenvironment

**FIGURE 8:**
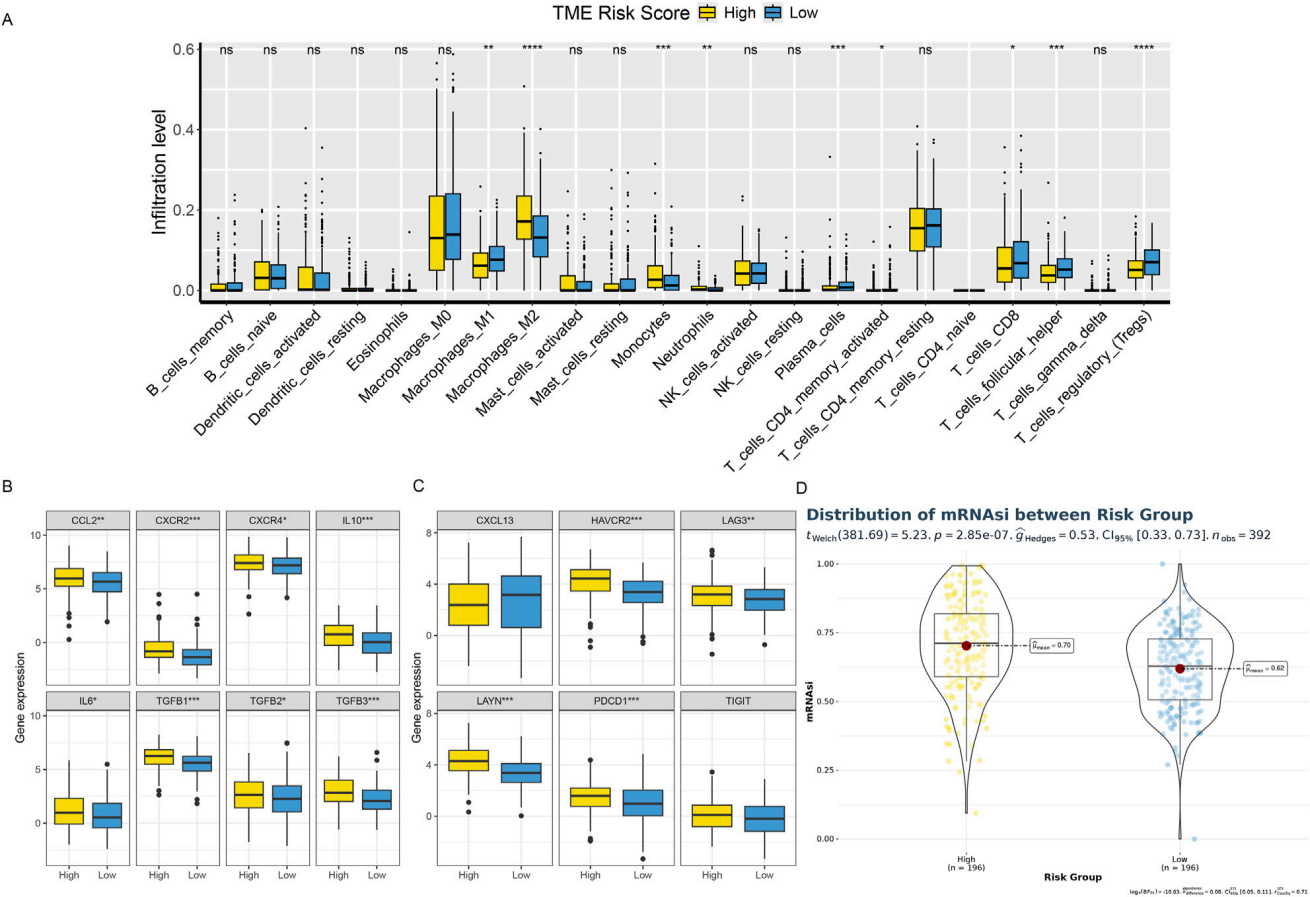
Shows the TME phenotypes in various risk categories. **(A)** Box plot showing the distributions across two risk categories of 22 immune cell subsets found by CIBERSORT. **(B, C)** The box figure illustrates the TEXterm characteristics and M2 polarization regulators expression patterns in two risk groups. **(D)** A violin plot comparing the two risk groups’ mRNAsi index values.

Based on the CIBERSORT immune microenvironment analysis algorithm, we analyzed the TCGA dataset divided into high-risk and low-risk groups. We found that the low-risk group had higher M1 macrophages and CD8^+^ T cell infiltration abundances, while the high-risk group had significantly higher abundances of M2 macrophage infiltration (Figure 8A). This suggests an active anti-tumor immune response in the tissue immunological microenvironment of the high-risk group. Further analysis of T cell exhaustion markers and M2 macrophage markers showed that the high-risk group had significantly higher expression levels than the low-risk group (Figures 8B, C). Simultaneously, analysis of cellular stemness levels also indicated higher stemness scores in the high-risk group, suggesting more pronounced tumor stemness, i.e., dedifferentiation (Figure 8D). Since cellular stemness levels are negatively correlated with prognosis, these results imply a poorer prognosis for patients in the high-risk group.

### 3.6 Relationship between risk score and responsiveness to drug treatment

**FIGURE 9:**
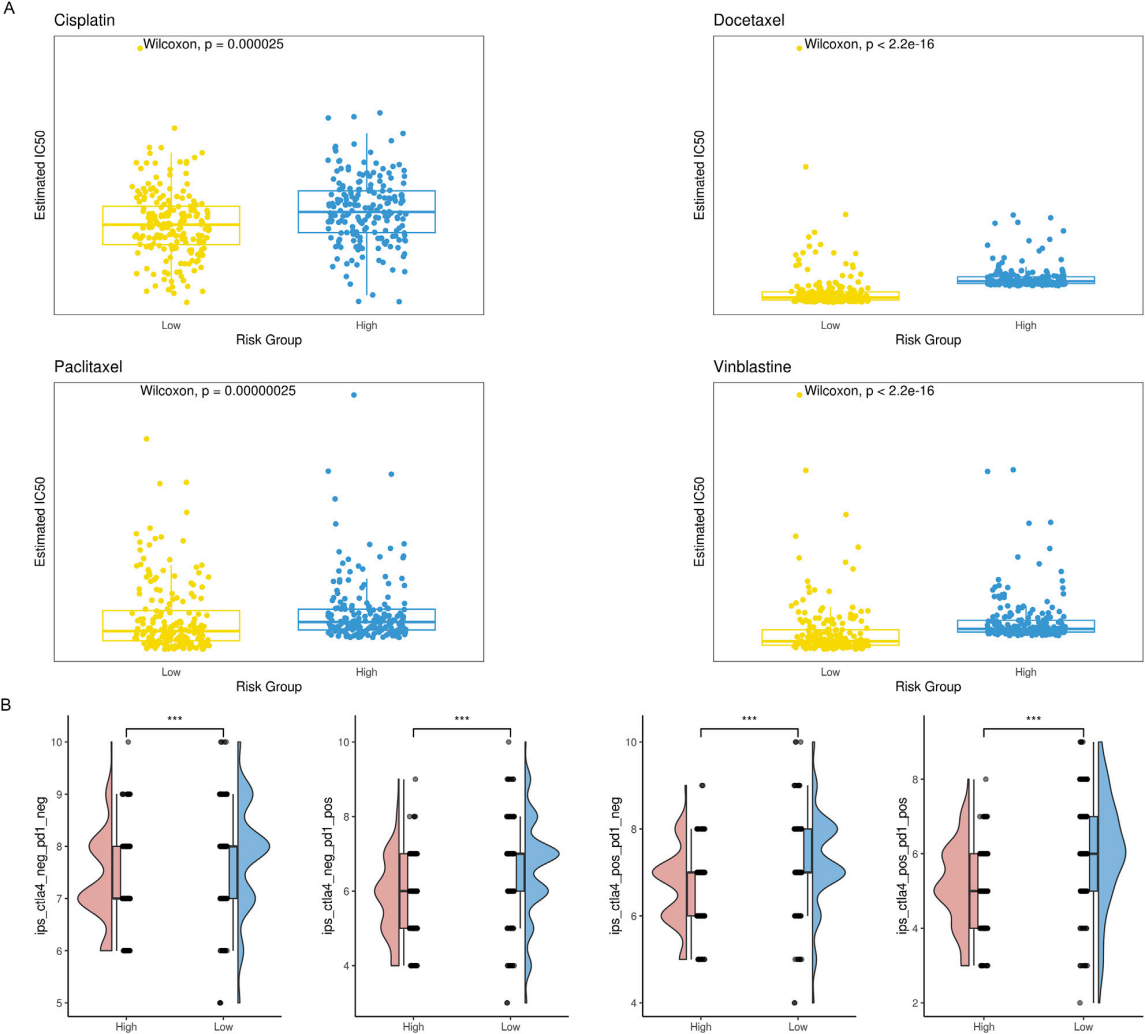
Comparison of therapeutic sensitivity between two risk categories. **(A)** Violin plot illustrating the expected IC50 values of therapeutic drugs for two different risk groups. **(B)** Raincloud plot illustrating two defined risk groups’ IPS scores.

The amount of immune cell infiltration substantially impacts immunotherapy and chemotherapy, according to previous studies, with the high-risk group having less anti-tumor immune cell infiltration than the low-risk group. To determine if the high-risk group responded less frequently to these four drugs, we examined the IC50 values of cisplatin, vinblastine, paclitaxel, and docetaxel. Compared to the low-risk group, the IC50 values of the high-risk group were much greater (Figure 9A). We employed IPS, IPS-PD1/PD-L1/PD-L2, IPS-CTLA4, and IPS-PD1/PD-L1/PD-L2 + CTLA4 simultaneously to evaluate variations in immune checkpoint inhibitor therapy response rates across various risk groups. IPS, IPS-PD1/PD-L1/PD-L2, and IPS-CTLA4 levels were considerably lower in the high-risk group than in the low-risk group (Figure 9B).

### 3.7 Relationship between risk score and cancer hallmark signaling pathways

**FIGURE 10:**
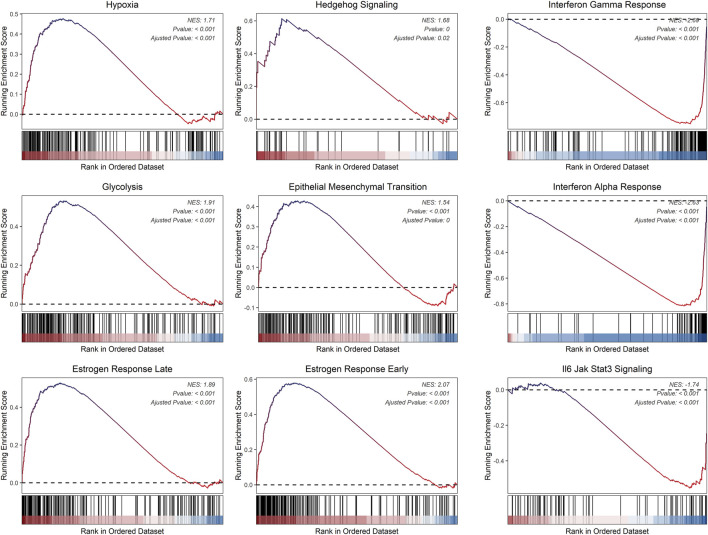
Hallmarks of dysregulated cancer in two risk groups.

We hypothesize that the distinct immune infiltration microenvironments and drug responsiveness observed among risk groups are based on significantly different signaling pathways and biological differences (Zeng et al., 2021). Consequently, we initially employed the limma R package to find genes differently expressed amongst various risk groups. Then, using cancer hallmarks as a guide, we ran a GSEA analysis on these differentially expressed genes. According to the findings, the high-risk group had significantly higher levels of several signaling pathways and significantly lower levels of others, including IL6-JAK-STAT3, hypoxia, and glycolysis. In summary, the prognostic model based on inflammatory genes exhibits distinct immune infiltration microenvironments, drug responsiveness, and deregulated signaling pathways, which has important implications for clinical decision-making (Figure 10).

### 3.8 Single-cell level analysis of risk scores

**FIGURE 11:**
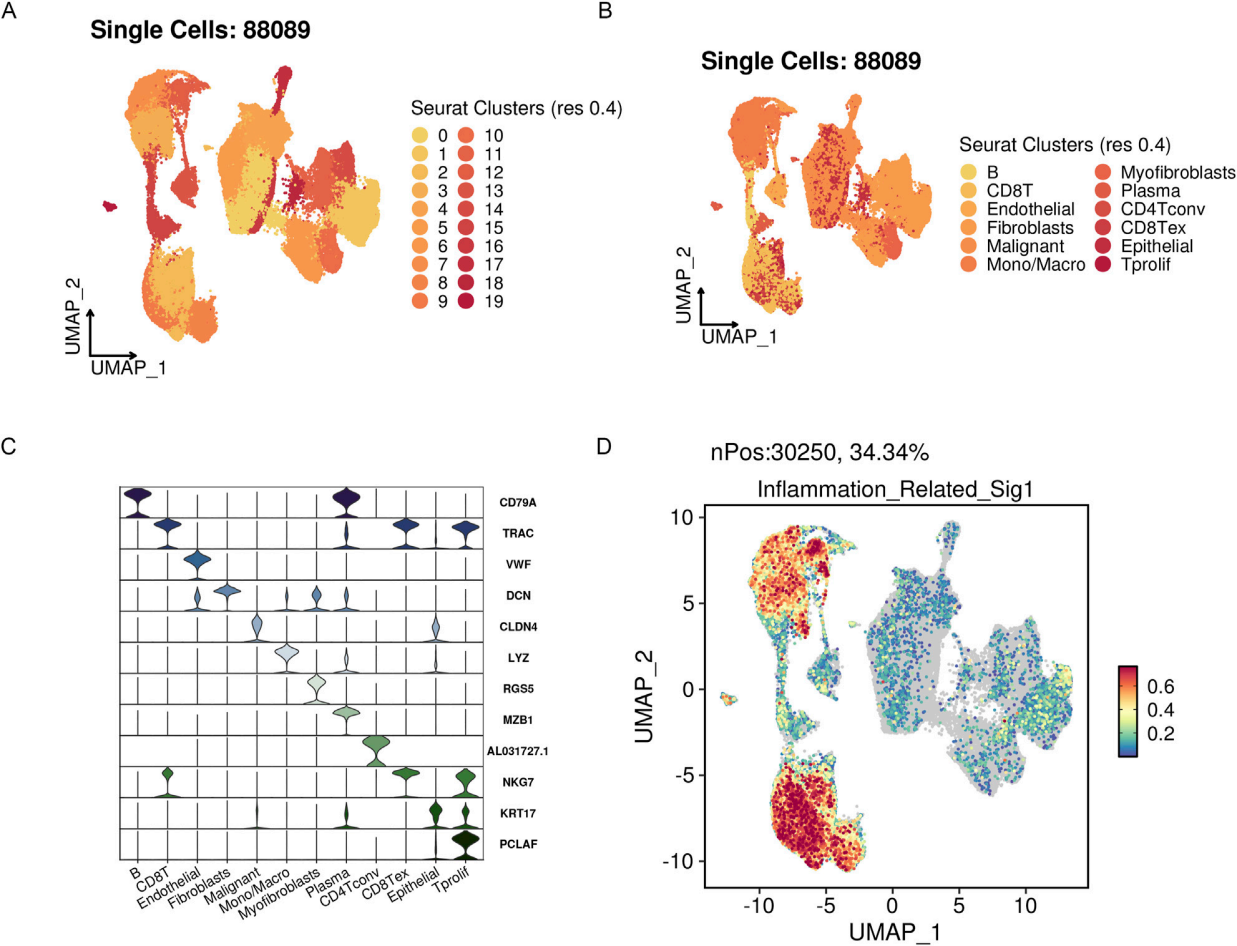
The highly active signature associated with inflammation in OV’s scRNA-seq datasets. **(A)** UMAP visualization of three public OV scRNA-seq cohorts with 88,089 cells. **(B)** A manual annotation was done on 12 major cell types. **(C)** Vlnplots showing cell type-specific marker expression values. **(D)** Single-cell signature gene expression is determined via Seurat’s AddModuleScore method.

We integrated four single-cell datasets and obtained 88,089 ovarian cancer single cells after quality control, dimensionality reduction, and clustering. With a clustering resolution set to 0.4, we identified 20 clusters (Figures 11A, B). Out of these 20 clusters, we identified 12 cell subpopulations by combining manual annotation methods with SingleR automatic annotation. We then analyzed the risk scores of these 12 cell subpopulations (Figures 11C, D). The results showed that the prognostic model genes related to inflammation were primarily expressed in immune-related cells, further validating the findings from traditional transcriptome analysis at the single-cell level.

### 3.9 Upregulation of IL6 and TGFβ1 in ovarian cancer cells promotes cell proliferation

**FIGURE 12:**
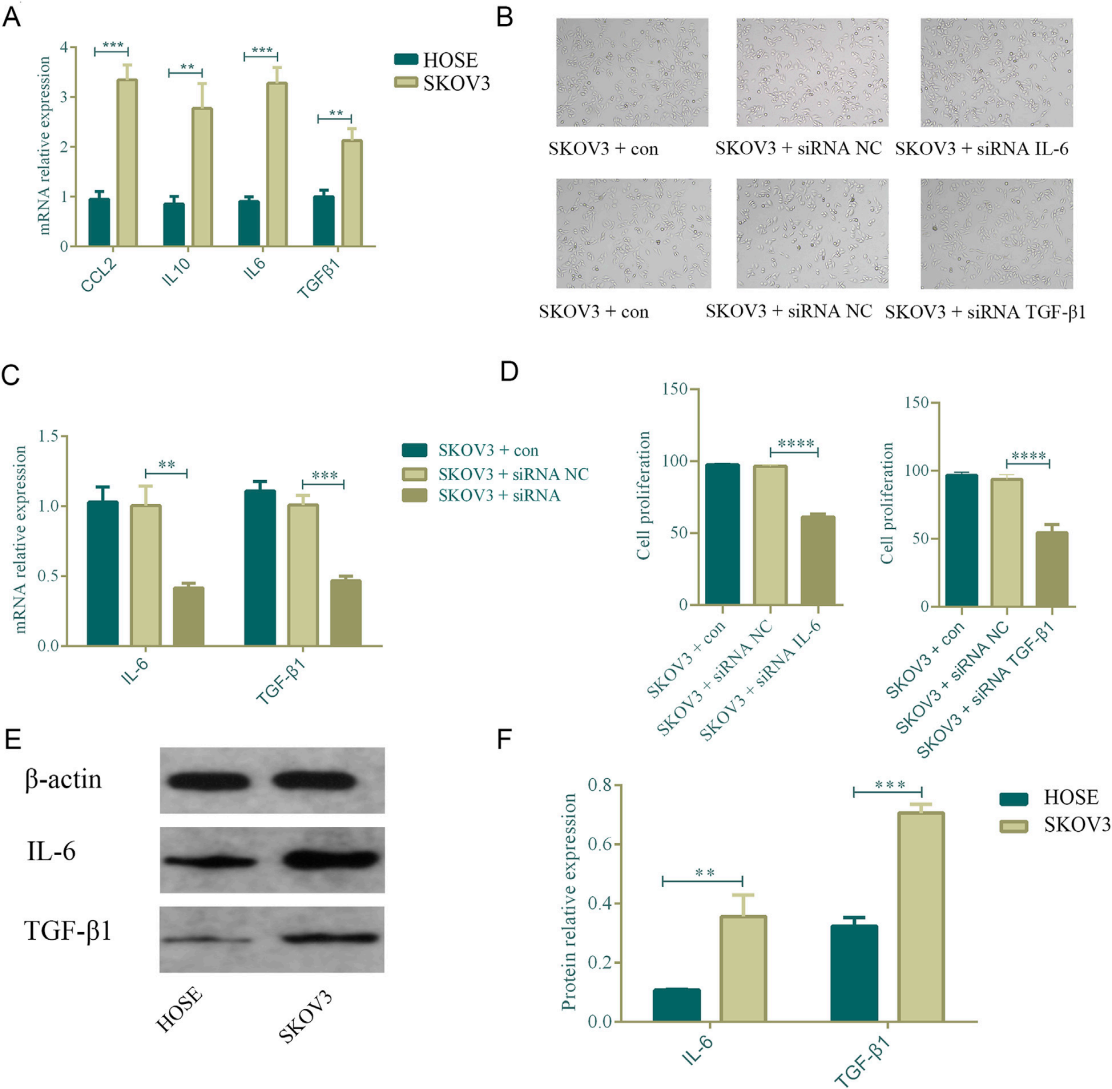
Upregulation of IL-6 and TGFβ1 in ovarian cancer cells promotes cell proliferation. n = 3. **p < 0.01, *p < 0.05, ****p < 0.0001, ***p < 0.001. **(A)** Comparison of the mRNA expression of CCL2, IL6, IL10, and TGFβ1 in normal ovarian cells and ovarian cancer cell SKOV3. **(B–D)** After knocking down IL6 and TGF-β1, the proliferation ability of ovarian cancer cells decreased. **(E,F)** Comparison of the protein expression of IL6 and TGFβ1 in normal ovarian cells and ovarian cancer cell SKOV3.

Following the bioinformatics analysis, the study explored the expression levels of CCL2, IL10, IL6, and TGFβ1 in normal ovarian epithelial cells (HOSE) and ovarian cancer cells (SKOV3). The research team used qPCR to detect the mRNA expression levels in cells. Compared with HOSE, CCL2 (p = 0.003), IL10 (p = 0.003), IL6 (p = 0.002), and TGFβ1 (p = 0.002) were all highly expressed in SKOV3, and the differences were statistically significant (Figure 12A). Subsequently, the study used siRNA IL6 and TGFβ1 to transfect the SKOV3 cell line. qPCR results revealed that following transfection, there was a drop in the mRNA expression levels of TGFβ1 (p = 0.0002) and IL6 (p = 0.002) (Figures 12B, C). Further, CCK8 was used to evaluate the proliferation of SKOV3 after transfection. Following the knockdown of IL6 and TGFβ1, the capacity of SKOV3 cells to proliferate decreased in comparison to the siRNA NC group. There was a statistically significant difference (p < 0.0001, p < 0.0001) (Figure 12D). Finally, we used Western blot to further evaluate the protein expression levels of IL6 and TGFβ1 in HOSE and SKOV3 cells. The SKOV3 group had higher levels of TGFβ1 (p = 0.0001) and IL6 (p = 0.004) protein expression compared to HOSE, and these changes were statistically significant (Figures 12E, F).

## 4 Discussion

Chemotherapy resistance in EOC results from various processes, including reduced drug sensitivity, the influence of the tumor microenvironment (TME), changes in the metabolism of tumor cells, interactions between stromal cells and tumor cells, and immune evasion mechanisms (Veneziani et al., 2023). Among these, the TME and immune evasion mechanisms play crucial roles in chemotherapy resistance in EOC. The TME has a major impact on drug resistance, metastasis, and tumor growth. It comprises stromal cells, immune cells, and blood vessels surrounding tumor cells (Agarwal and Kaye, 2003). In ovarian cancer, the tumor microenvironment can promote drug resistance through various mechanisms (Pujade-Lauraine et al., 2019). For example, tumor-associated macrophages (TAMs) can secrete multiple growth factors and inflammatory cytokines, promoting tumor cell proliferation and survival while reducing their sensitivity to chemotherapy drugs. Immune evasion mechanisms (Khan et al., 2021; Kim et al., 2012): Although tumor cells can employ several defence mechanisms to evade immune system attacks, which is crucial to antitumor processes. For instance, tumor cells can express immune checkpoint molecules to inhibit T-cell activity or secrete immunosuppressive factors to suppress the proliferation and function of immune cells.

Several studies have shown that inflammatory oxidative stress responses play a role in the pathogenesis of several cancers, including colon, stomach, and liver (Bast et al., 1993; Ray et al., 2023). Phagocytes and leukocytes recruited during inflammatory responses can induce DNA damage by producing peroxides and reactive nitrogen species, leading to gene mutations, deletions, and rearrangements, which in turn cause tumorigenesis. The massive secretion of pro-inflammatory factors is a hallmark of chronic inflammatory response processes and plays a vital role in the development of tumors. For example, IL-6 reduces the expression of tumor suppressor genes and DNA repair genes by inducing DNA methylation, thereby promoting tumorigenesis (Macciò and Madeddu, 2013; Torres et al., 2009). IL-6 and its downstream targets are closely related to processes such as cell proliferation and metabolism, suggesting its contribution to tumorigenesis. The expression of the proto-oncogene Kras in the pancreas activates the Stat3/Socs3 signaling pathway, which relies on IL-6 and its downstream signaling pathways, ultimately promoting pancreatic cancer development. Inflammatory cytokines released during inflammatory responses facilitate tumor metastasis and invasion. Epithelial-mesenchymal transition (EMT) of tumor cells is a crucial process for their metastasis and invasion. TGFβ (Monavarian et al., 2022; Brewer et al., 2003; Vergara et al., 2010) has been reported to promote EMT in tumor cells, while TNFα, IL-6, and IL-1 can also promote tumor ETM by upregulating gene expression related to transcription factors such as NF-κB and STAT3. Additionally, pro-inflammatory factors upregulate chemokine receptors such as CCR1, CCR4, and CXCR7, enabling tumor cells to metastasize to specific organs. Therefore, inflammatory cytokines and mediators in the tumor microenvironment are essential for tumor cell survival, metastasis, and development. Our research has found that in the high-risk group of EOC (Macciò and Madeddu, 2012; Browning et al., 2018), various signaling pathways, including hypoxia and glycolysis, are significantly upregulated, while pathways such as IL6-JAK-STAT3 are downregulated. These findings further confirm the significance of targeting inflammatory genes to improve drug responses in the immune microenvironment.

Moreover, in the risk model based on inflammatory gene scoring, the high-risk group exhibits higher infiltration of M2 macrophages with pronounced anti-tumor immune responses and a higher degree of dedifferentiation. Additionally, patients in the high-risk group show significantly lower response rates to IPS, IPS-PD1/PD-L1/PD-L2, and IPS-CTLA4 inhibitors compared to the low-risk group. Single-cell transcriptome sequencing data confirms this, with inflammatory prognosis model genes primarily expressed in immune-related cells. In the tumor microenvironment of ovarian cancer, the immune cells comprise innate and adaptive immune cells. B lymphocytes and T lymphocytes are components of the adaptive immune system, with T lymphocytes being particularly prevalent in ovarian tumor tissue and ascites (Fucikova et al., 2022; McMullen et al., 2021). Tumor-infiltrating lymphocytes (TILs) are T cells found in primary/metastatic tumors; tumor-associated lymphocytes (TALs) are T cells seen in ascites (Cummings et al., 2021). Through suppressing immune responses, CD4^+^ Tregs preserve immune homeostasis and promote self-tolerance. Tregs inhibit anti-tumor responses in cancers, and their presence in the ovarian cancer tumor microenvironment has been associated with a poor prognosis. The loss of human leukocyte antigen (HLA)-I expression by tumor cells is the primary mechanism of immune evasion in T cell-mediated anti-tumor immunity (Lavoué et al., 2013; Zhang et al., 2022). There is a direct correlation between the frequency of TILs and the quantity of HLA-I-positive tumor cells in various solid tumors, including ovarian cancer. T-cell exhaustion is another method of immune evasion. Studies have revealed that TILs and TALs exhibit elevated expression levels of ICRs for PD-1, CTLA-4, TIM-3, BTLA, and LAG-3.

Tumor macrophages, in addition, comprise a very diverse and heterogeneous cell population that can be divided into type 2 (M2) and classically activated type 1 (M1) macrophages. The microenvironment of ovarian cancer tumors is rich in IL-6, IL-10, and CSF-1, which promotes M2 polarization and the accumulation of M2 macrophages (Truxova et al., 2023). An increase in the proportion of M2 macrophages often indicates a poor prognosis in ovarian cancer. These data imply that tumor macrophages may stimulate tumor growth, invasion, and metastasis via various pathways. The extracellular matrix (ECM) is another component of the immune microenvironment in addition to immune cells (Lin et al., 2022; Khatoon et al., 2022), which affects tumor growth and metastasis. Studies have shown that ECM affects cancer cells via biochemical and biophysical mechanisms in addition to acting as a physical structure and growth factor reservoir. Activating the signaling pathways for ERK, PI3K, and Rac; changing the function of cell cycle regulatory elements; regulating pro- and anti-apoptotic regulators (Bcl-2 and NF-κB); influencing tumor invasion and migration through the signaling pathways for TGFβ and RhoA/Rac; influencing tumor cell stemness through the activation of STAT3 and Wnt; and activating the previously mentioned anti-apoptotic and stem cell signaling pathways, in addition to acting as a physical barrier to the delivery of anticancer drugs, which results in chemotherapy resistance (De Nola et al., 2019; Rodriguez et al., 2018; Damei et al., 2023; Majidpoor and Mortezaee, 2021).

Numerous studies have demonstrated the complex interplay between inflammation, the immune microenvironment, and the development and progression of EOC. Our findings imply that by using genes linked to inflammation, a better understanding of the characteristics of the immunological milieu in EOC patients can be achieved. This approach can facilitate the development of targeted immunotherapy drugs for different risk groups, ultimately improving patient prognosis.

Although the study comprehensively analyzed multiple data sets related to ovarian cancer, the sample size is still very limited. Patients of different races, regions, and genetic backgrounds may have different molecular characteristics and immune response mechanisms, and may not fully represent the diversity of ovarian cancer patients worldwide. In addition, bioinformatics tools play an important role in gene expression analysis, mutation detection, and pathway enrichment, but these tools themselves have limitations. Although *in vitro* experiments are an important means to verify gene function and pathway activity, the *in vitro* environment cannot fully simulate the complexity of the *in vivo* environment. Therefore, the results of *in vitro* experiments may not be directly applicable to the *in vivo* environment, and subsequent studies need to be further verified in animals and multi-center clinical samples.

## 5 Conclusion

In conclusion, the construction of an ovarian cancer prognosis model based on inflammatory-related prognostic genes can stratify EOC patients by risk. Developing corresponding drugs based on the characteristics of the immune infiltration environment, drug responsiveness, and signaling pathways of different risk groups is of great significance for clinical decision-making.

## Data Availability

The original contributions presented in the study are included in the article/supplementary material, further inquiries can be directed to the corresponding authors.
